# Perceptually relevant speech tracking in auditory and motor cortex reflects distinct linguistic features

**DOI:** 10.1371/journal.pbio.2004473

**Published:** 2018-03-12

**Authors:** Anne Keitel, Joachim Gross, Christoph Kayser

**Affiliations:** 1 Institute of Neuroscience and Psychology, University of Glasgow, Glasgow, United Kingdom; 2 Institute for Biomagnetism and Biosignalanalysis, University of Münster, Münster, Germany; 3 Cognitive Neuroscience, Bielefeld University, Bielefeld, Germany; University College London, United Kingdom of Great Britain and Northern Ireland

## Abstract

During online speech processing, our brain tracks the acoustic fluctuations in speech at different timescales. Previous research has focused on generic timescales (for example, delta or theta bands) that are assumed to map onto linguistic features such as prosody or syllables. However, given the high intersubject variability in speaking patterns, such a generic association between the timescales of brain activity and speech properties can be ambiguous. Here, we analyse speech tracking in source-localised magnetoencephalographic data by directly focusing on timescales extracted from statistical regularities in our speech material. This revealed widespread significant tracking at the timescales of phrases (0.6–1.3 Hz), words (1.8–3 Hz), syllables (2.8–4.8 Hz), and phonemes (8–12.4 Hz). Importantly, when examining its perceptual relevance, we found stronger tracking for correctly comprehended trials in the left premotor (PM) cortex at the phrasal scale as well as in left middle temporal cortex at the word scale. Control analyses using generic bands confirmed that these effects were specific to the speech regularities in our stimuli. Furthermore, we found that the phase at the phrasal timescale coupled to power at beta frequency (13–30 Hz) in motor areas. This cross-frequency coupling presumably reflects top-down temporal prediction in ongoing speech perception. Together, our results reveal specific functional and perceptually relevant roles of distinct tracking and cross-frequency processes along the auditory–motor pathway.

## Author summary

How we comprehend speech—and how the brain encodes information from a continuous speech stream—is of interest for neuroscience, linguistics, and research on language disorders. Previous work that examined dynamic brain activity has addressed the issue of comprehension only indirectly, by contrasting intelligible speech with unintelligible speech or baseline activity. Recent work, however, suggests that brain areas can show similar stimulus-driven activity but differently contribute to perception or comprehension. To directly address the perceptual relevance of dynamic brain activity for speech encoding, we used a straightforward, single-trial comprehension measure. Furthermore, previous work has been vague regarding the analysed timescales. We therefore base our analysis directly on the timescales of phrases, words, syllables, and phonemes of our speech stimuli. By incorporating these two conceptual innovations, we demonstrate that different areas of the brain track acoustic information at the time-scales of words and phrases. Moreover, our results suggest that the motor cortex uses a cross-frequency coupling mechanism to predict the timing of phrases in ongoing speech. Our findings suggest spatially and temporally distinct brain mechanisms that directly shape our comprehension.

Speech consists of hierarchically organised linguistic segments such as phrases, words, syllables, and phonemes [1,2]. To comprehend speech, a listener needs to parse the continuous stream into these segments [3]. One mechanism that has been proposed to fulfil such a role is the tracking of speech information in dynamic brain activity (often termed speech-to-brain entrainment) [4,5]. Such tracking is observed as a precise alignment of rhythmic brain activity to the temporal characteristics of speech. Previous studies commonly focused on brain activity at the timescales of traditional delta (1–4 Hz) and theta (4–8 Hz) bands [2,6,7,8]. These have been suggested to reflect the neural processing of prosodic and syllabic speech features [e.g., 3]. However, such a general association between timescales in brain activity and speech properties is difficult [9]. First, there are large interindividual differences in speaking rate and use of prosody. For example, the syllabic rate (sometimes interchangeably used with speech modulation rate) has been found within a range of 2 to 20 Hz across studies [10,11,12,13]. This indicates that the syllabic rate does not always fall into the often used theta frequency band. Second, the association between specific timescales and linguistic or phonological features remains ambiguous because multiple properties have been associated with delta (stress, intonation, phrase structure) [14,15,16] and theta bands (syllables, jaw movements) [17,18]. In the present study, we therefore take a different approach and first extract linguistic timescales from the speech corpus, based on stimulus-specific regularities. We then use these to investigate the neural mechanisms underlying speech encoding. We believe that any observed neural or perceptual effects at these linguistically specific timescales allow a more straightforward and mechanistically more specific interpretation than effects at generic timescales.

The functional interpretation of speech-to-brain entrainment is further hampered by a lack of a systematic assessment of where (in the brain) and at which timescale it is relevant for a perceptual outcome. By combining neural recordings with a behavioural measure, it has recently been shown that much distributed neural activity represents processes contributing to sensory encoding and perception, and only some focal activity is causally related to perceptual decisions [19,20]. Brain areas can therefore have similar stimulus-driven responses but different causal relationships with perceptual decisions [20]. By contrast, most studies attempting to link comprehension and speech tracking have done so only indirectly (i.e., without probing perceptual decisions) (but see [21]). These studies typically vary speech intelligibility by using background noise [22,23], noise vocoding [7,24], reversed speech [2,25,26,27], or by shuffling syllables [28]. Thereby, they have revealed a link between neural delta and theta band tracking and intelligibility during listening to continuous speech [2,5,21]. Interestingly, some of these studies have demonstrated widespread low-frequency tracking across the brain, going beyond auditory cortex and encompassing prefrontal and motor areas [2,22]. However, because many studies contrast brain activity during actual speech encoding with a surrogate dataset reflecting no speech–brain relation, these only demonstrate the existence of a speech-to-brain encoding process but not its functional or perceptual relevance. This notion is also supported by the finding that auditory entrainment can be observed for subthreshold stimuli [29], suggesting that there is no necessary perceptual consequence of this tracking process. Therefore, it remains uncertain whether and where the tracking of speech by dynamic brain activity is perceptually relevant for comprehension at the single-trial level.

In the present study, participants performed a comprehension task on natural, structurally identical sentences embedded in noise. Sentences were semantically unpredictable, but meaningful. Using magnetoencephalography (MEG), we analysed speech tracking (quantified by mutual information [MI]) in source-localised data at the single-trial level. We then directly tested the perceptual relevance of speech tracking at timescales mapping onto linguistic categories such as phrasal elements, words, syllables, and phonemes.

## Results

### Behavioural results

Participants listened to single sentences and indicated after each trial which (out of 4) target words occurred in the sentence. Participants reported the correct target word, on average, in 69.7 ± 7.1% (mean ± SD) of trials, with chance level being 25%. Performance of individual participants ranged from 56.1% to 81.1%, allowing a comparison between correct and incorrect trials for each participant.

### Overall speech tracking

**Fig 1:**
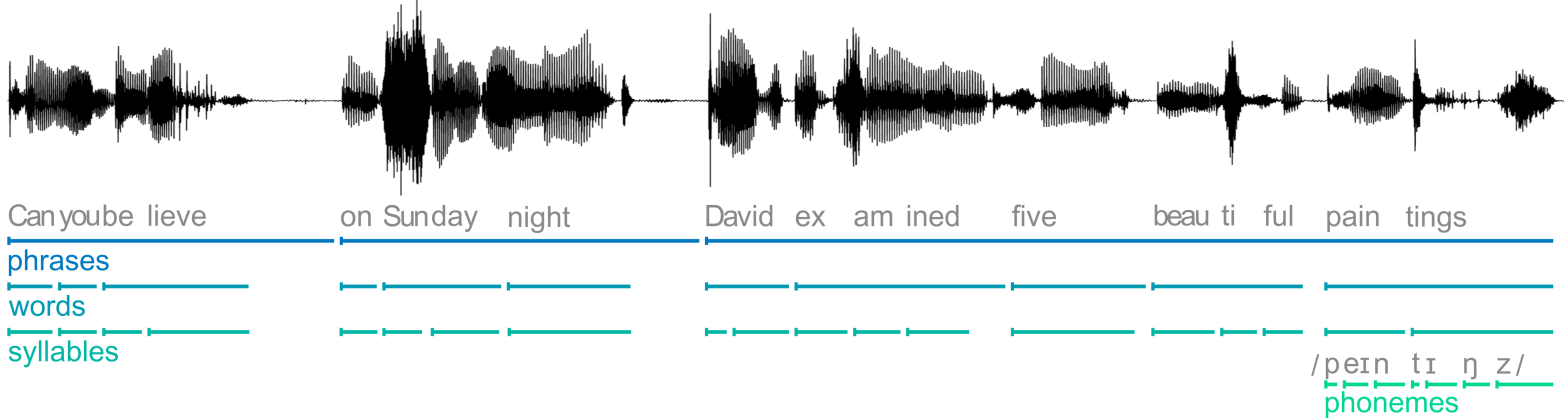
Example sentence from the stimulus material. Shown is the acoustic waveform (black line) as well as its segmentation into phrases, words, syllables, and phonemes (last word only). Example sentence deposited in the Dryad repository: https://doi.org/10.5061/dryad.1qq7050 [31].

**Fig 2:**
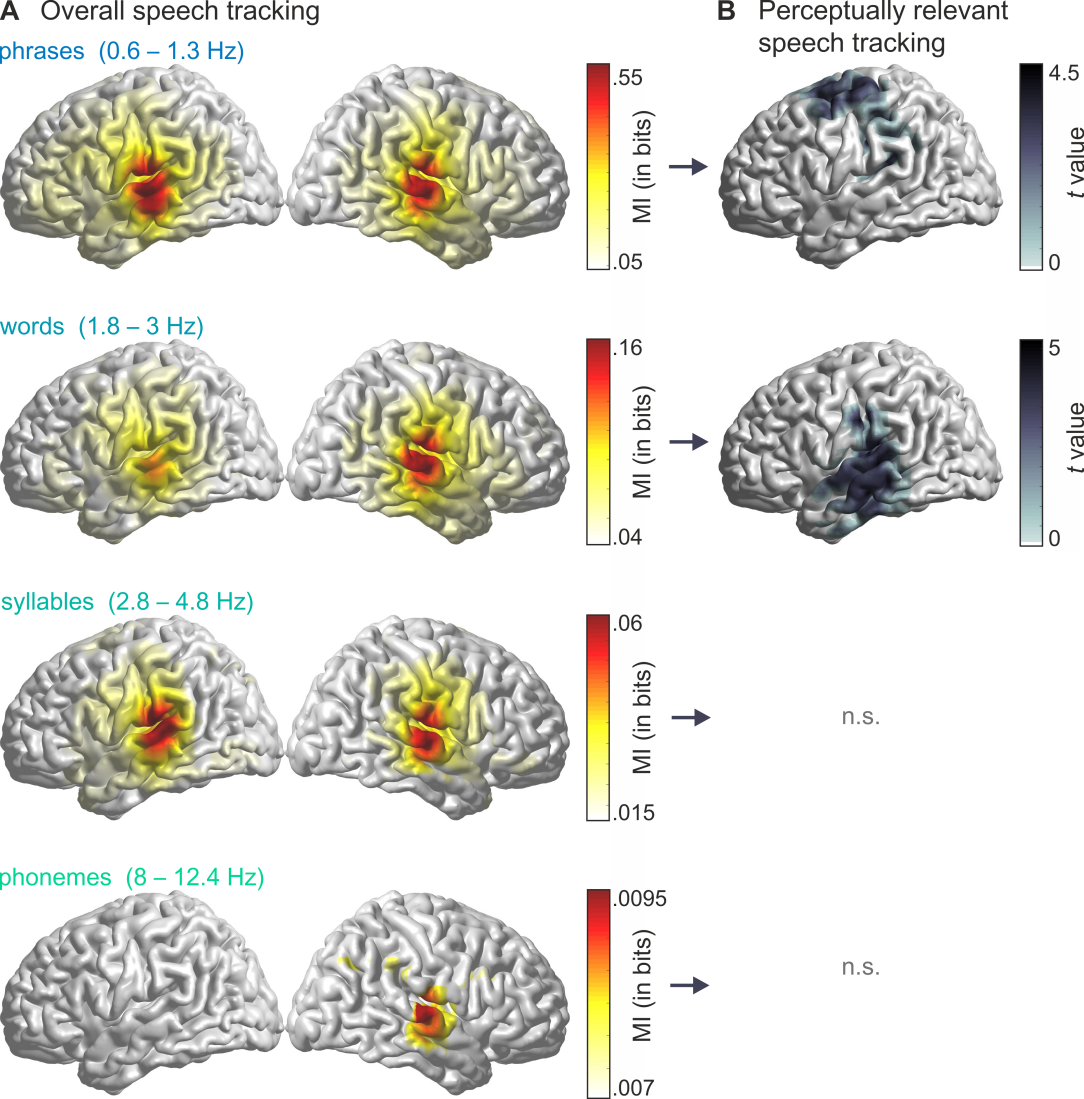
Overall and perceptually relevant speech tracking in the 4 stimulus-tailored frequency bands. (A) Significant areas for comparison between true MI values and surrogate data (*t* test, cluster-corrected). The used frequency bands map onto timescales for phrases (0.6–1.3 Hz), words (1.8–3 Hz), syllables (2.8–4.8 Hz), and phonemes (8–12.4 Hz). (B) Clusters where speech tracking MI was larger for correctly comprehended compared to incorrectly comprehended trials (*t* test, cluster-corrected). Data deposited in the Dryad repository: https://doi.org/10.5061/dryad.1qq7050 [31]. MI, mutual information; n.s., not significant.

The MI between the source-localised, Hilbert-transformed MEG time series and the Hilbert-transformed speech envelope was computed within 4 frequency bands. These reflected the rates of phrases (0.6–1.3 Hz), words (1.8–3 Hz), syllables (2.8–4.8 Hz), and phonemes (8–12.4 Hz) in the stimulus corpus (for an example sentence, see Fig 1). The boundaries of each band were defined based on the slowest and fastest event per linguistic category across sentences (see Materials and methods). When compared to surrogate data, speech MI was significant in all analysed frequency bands (Fig 2A; phrases: *T*_sum_(19) = 32,262.9, *p* < .001; words: *T*_sum_(19) = 22,2243.8, *p* < .001; syllables: *T*_sum_(19) = 13,428.6, *p* < .001; phonemes: *T*_sum_(19) = 1,294.0, *p* = .018). As in previous studies, MI was strongest in early auditory areas [2,22,30] and decreased with increasing frequency [6,22]. Tracking of phrases, words, and syllables was reflected in a bilateral cluster, whereas phoneme tracking was only significant in the right hemisphere. These results confirm the previously reported existence of speech encoding in rhythmic brain activity versus a null hypothesis of no encoding but do not speak on the perceptual relevance.

### Perceptually relevant speech tracking

**Fig 3:**
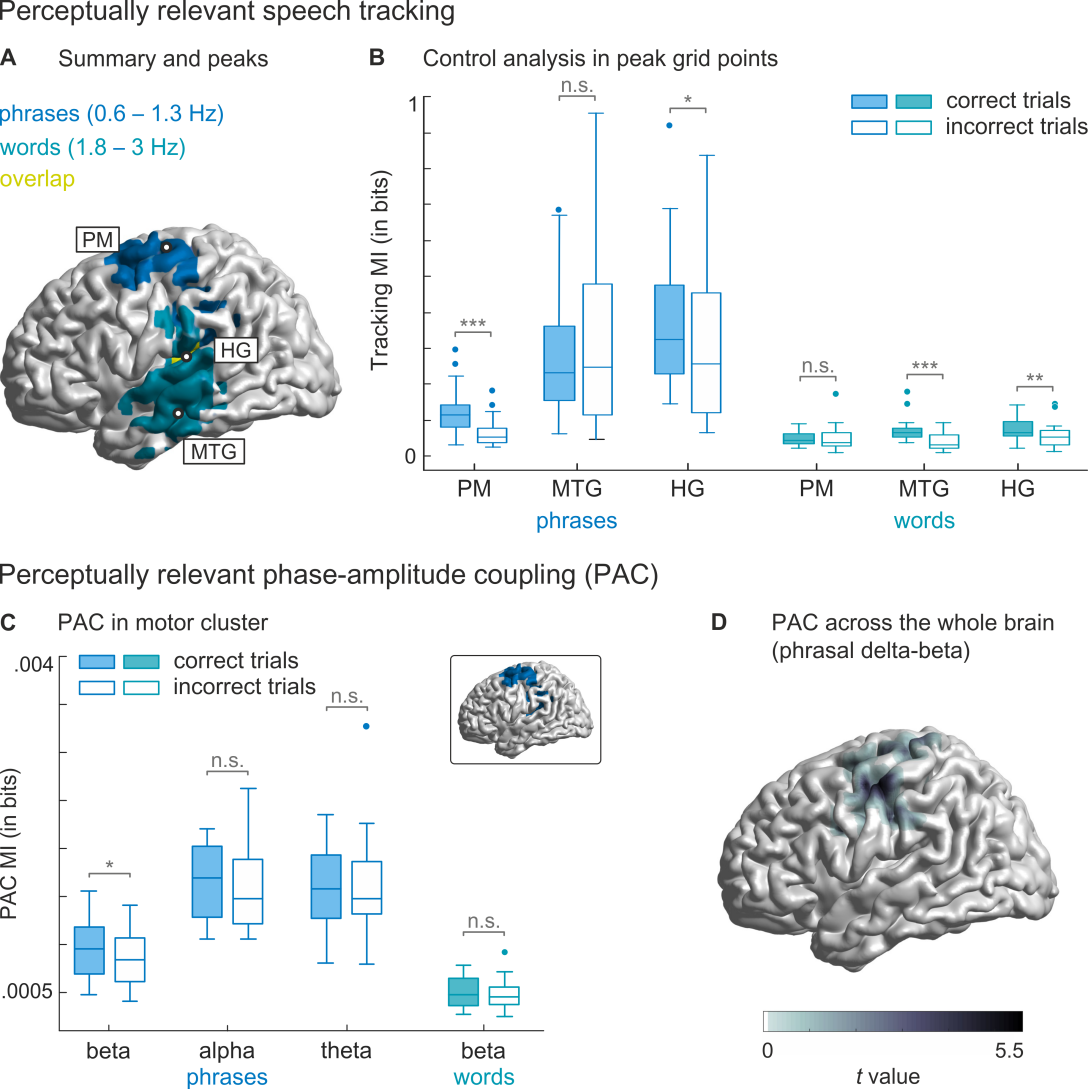
Summary of perceptually relevant effects and control analyses. (A) Brain regions with perceptually relevant speech tracking. For phrases (blue), the effect was strongest in the left PM area. For words (turquoise), it was strongest in the left MTG. Areas that were perceptually relevant for both phrases and words (yellow) include left HG and supramarginal gyrus. Peak grid points are denoted with circles. (B) Comparison of tracking MI values at peak grid points in PM cortex, MTG, and HG for phrase and word scales. Boxes denote interquartile range with median line; error bars show minimum and maximum, excluding outliers. (C) MI between the phase at the phrasal timescale (0.6–1.3 Hz) and power in beta, alpha, and theta bands (blue plots), as well as the phase at the word timescale (1.8–3 Hz) and beta power (turquoise plot) for correctly and incorrectly comprehended trials, averaged across all grid points in the motor cluster (cluster shown in inlet). Only PAC between the phase at the phrasal timescale and beta power was perceptually relevant. (D) Whole-brain analysis across all 12,337 grid points confirming that PAC between phrasal phase and beta power is indeed confined to motor regions. Coloured area denotes the cluster where PAC between phrasal phase and beta power was larger for correctly comprehended than uncomprehended trials. Significance in panel B and C is denoted with: *** = *p* < .001, ** = *p* < .01, * = *p* < .05. Data deposited in the Dryad repository: https://doi.org/10.5061/dryad.1qq7050 [31]. HG, Heschl gyrus; MI, mutual information; MTG, middle temporal gyrus; n.s., not significant; PAC, phase-amplitude coupling; PM, premotor.

To localise cortical regions where entrainment was functionally relevant for comprehension, we statistically compared MI between correct and incorrect trials within each band (hereafter called ‘perceptually relevant’). This yielded significant left-hemispheric clusters in 2 frequency bands (Fig 2B). For the phrasal timescale (0.6–1.3 Hz), MI was larger for correctly versus incorrectly comprehended trials in a cluster comprising left pre- and postcentral regions, supramarginal gyrus, and Heschl gyrus (HG; *T*_sum_(19) = 568.00, *p*_cluster_ = .045; 205 grid points). The effect peaked in the left premotor (PM) cortex (left Brodmann area [BA] 6). For the word timescale (1.8–3 Hz), MI was larger in a cluster comprising left superior, middle, and inferior temporal gyrus as well as supramarginal gyrus and HG (*T*_sum_(19) = 739.59, *p*_cluster_ = .018; 263 grid points). The effect peaked in the left middle temporal gyrus (MTG; left BA 21). There was a small overlap of the clusters for phrasal and word timescales, peaking in the left HG (left BA 41, see Fig 3A).

We performed several further analyses to determine whether these effects were specific to those timescales extracted from the stimulus corpus. First, we performed posthoc *t* tests at the peak grid points of each cluster to see whether phrasal and word effects were indeed significant only for the respective timescales (Fig 3B). As expected, MI differed between correct and incorrect trials at the phrasal timescale in left PM cortex and HG (*t*(19) = 4.90, *p*_FDR_ < .001 and *t*(19) = 2.53, *p*_FDR_ = .031, respectively). Likewise, MI differed at the word timescale in the left MTG and HG (*t*(19) = 5.22, *p*_FDR_ < .001 and *t*(19) = 3.48, *p*_FDR_ = .005, respectively). MI neither differed at the phrasal scale in MTG (*t*(19) = −1.78, *p*_FDR_ = .11) nor at the word scale in PM cortex (*t*(19) = −0.57, *p*_FDR_ = .58). We also compared correct and incorrect trials at the same peak grid points for syllable and phoneme timescales, although the whole-brain analysis did not indicate that effects were perceptually relevant for the task at these timescales. This was to make sure that effects had not been overlooked due to corrections for multiple comparisons. None of the comparisons were significant (all *p*_FDR_ > .56, see S1 Fig), indicating that none of the peak grid points in PM, HG, or MTG showed perceptual relevance at the faster scales of syllables or phonemes.

Second, we compared brain-wide MI values between correctly and incorrectly comprehended trials in 7 generic, 2 Hz–wide frequency bands (from 0–8 Hz, in 1-Hz steps) to confirm that the above intelligibility-related effects were indeed specific to frequency bands matched to the specific temporal structure of the speech material. This is an important contrast because most previous studies used generic bands with a predefined fixed frequency spacing. Perceptually relevant effects were found in only 2 bands (S2A Fig). For the 1–3 Hz band, which largely overlaps with the word scale, MI was larger for comprehended than uncomprehended trials in a cluster centred around auditory cortex (*T*_sum_(19) = 1,078.85, *p*_cluster_ = .030), confirming the relevance of auditory regions for word-level encoding. For the 2–4 Hz band, which spans the scale of words and syllables, MI was only marginally enhanced for comprehended sentences in a cluster covering middle and inferior temporal cortex (*T*_sum_(19) = 751.93, *p*_cluster_ = .046).

Third, using posthoc statistics, we also verified that the MI at the previously identified peak grid points (see Fig 3A for peaks) differed between correct and incorrect trials only at those timescales that matched the stimulus-specific bands (S2B Fig). The motor cortex was not found to be perceptually relevant in any of the probed generic bands, which suggests that exact frequency boundaries are necessary to detect phrase tracking in motor areas.

### Phase-amplitude coupling in motor cortex

Rhythmic brain activity represents neuronal excitability changes [32,33]. In auditory areas, this mechanism has been suggested to reflect a segmentation of the incoming sensory stream [34,35]. But what is the role of slow excitability changes in the motor system? The motor system plays a role in the temporal prediction of rhythms and beats [36,37,38,39]. Previous studies have suggested that these predictions rely on the coupling of delta phase to rhythmic activity in the beta band [36,40,41]. We therefore hypothesised that speech entrainment at the phrasal scale—and its perceptual relevance—is directly linked to phase-amplitude coupling (PAC) with motor cortical beta activity. Indeed, we found that the coupling of the phase of phrasal-scale activity (0.6–1.3 Hz) to beta power (13–30 Hz) was significantly stronger for correctly versus incorrectly comprehended trials in our motor cluster (*t*(19) = 2.96, *p*_FDR_ = .032; Fig 3C). In contrast, there was no such cross-frequency coupling for the phase of word-scale activity relative to beta power (*t*(19) = 1.14, *p*_FDR_ = .356) or of phrasal phase to either alpha (*t*(19) = 1.38, *p*_FDR_ = .356) or theta power (*t*(19) = −0.38, *p*_FDR_ = .708).

To confirm that the PAC between phrasal phase and beta power is confined to motor regions, we performed a further whole-brain analysis, comparing PAC between correct and incorrect trials. This analysis yielded 1 cluster in which PAC was larger for comprehended than uncomprehended trials (*T*_sum_ = 203.94, *p*_cluster_ = .030, one-sided; Fig 3D). The cluster included left pre- and postcentral regions. Therefore, perceptually relevant PAC was confined to the left motor system, overlapping with the speech tracking effect in left motor areas.

### Phrasal tracking and PAC in additional dataset

**Fig 4:**
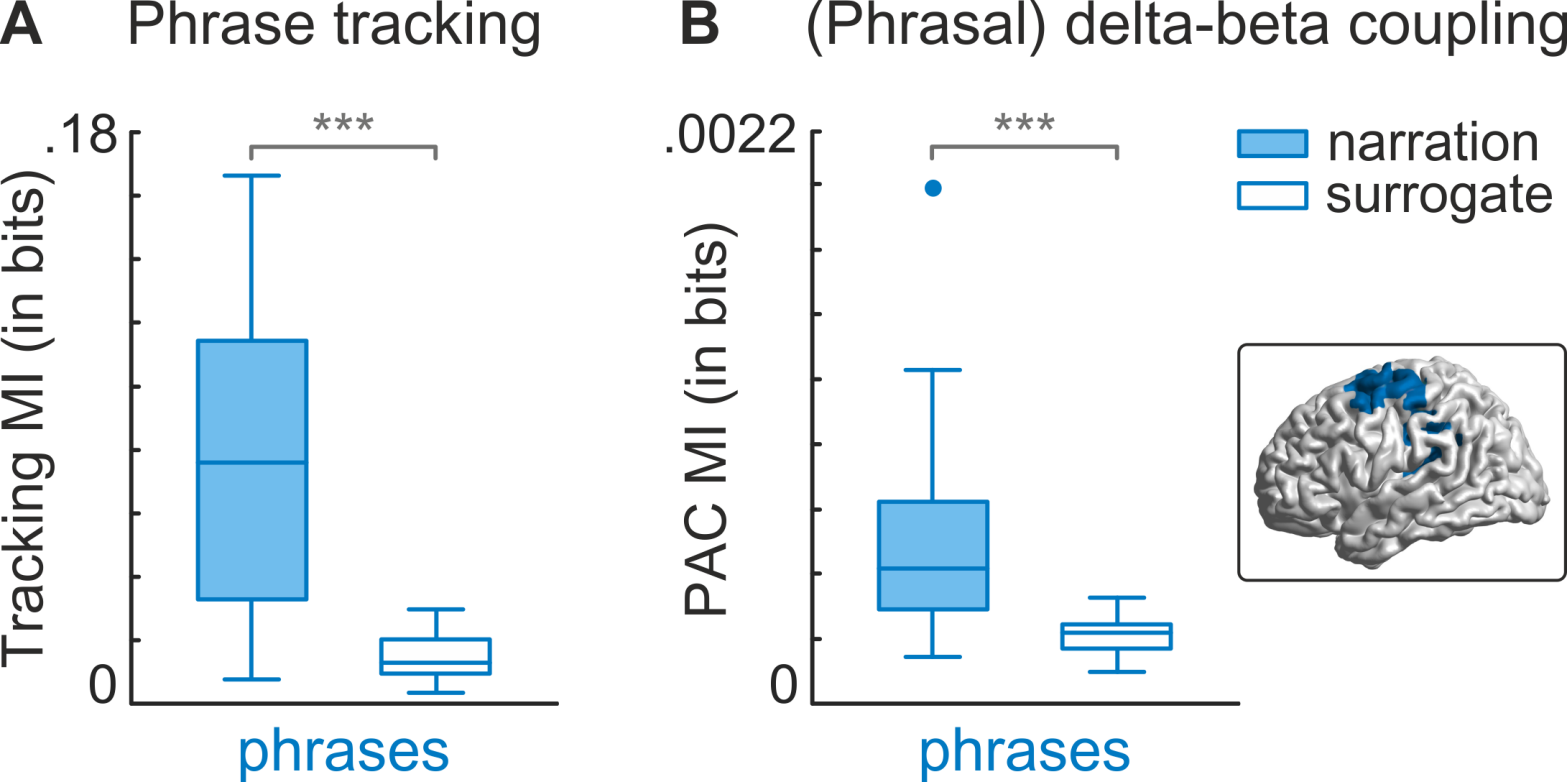
Phrase tracking and PAC in motor cortex in an additional dataset. MI values for phrase tracking and PAC were compared with surrogate data. Surrogate data were created by reversing the time series for speech and computing MI between forward brain time series and reversed speech time series. Surrogate data represent values that would be expected by chance. (A) Speech tracking at the phrasal time-scale (0.1–1.5 Hz) for real and surrogate data in the motor cortex (see inlet for analysed area). Phrase tracking was significantly larger in real data than in surrogate data. (B) MI between the phase at the phrasal timescale (0.1–1.5 Hz) and power in the beta band (13–30 Hz). PAC was significantly larger in real data than in surrogate data. Significance is denoted with: *** = *p* < .001. Data deposited in the Dryad repository: https://doi.org/10.5061/dryad.1qq7050 [31]. MI, mutual information; PAC, phase-amplitude coupling.

The sentence structure in the present study was relatively rigid and predictable, which could have emphasised effects at the phrasal timescale. We therefore tested the presence of the phrasal tracking effect and the PAC between phrasal phase and beta power in the motor cortex in an additional dataset in which the sentence and phrase structure were more variable. Here, participants listened to a natural 7-min narration while their MEG was recorded [2,42]. The phrasal rate of the narration ranged between 0.1 Hz and 1.5 Hz. We specifically tested phrase tracking and PAC in the motor cluster, defined by results in the main dataset above, by contrasting the actual MI with surrogate data. Phrase tracking was larger in actual data (*t*(22) = 6.22, *p*_FDR_ < .001), as was PAC between phrasal phase and beta power (*t*(22) = 4.52, *p*_FDR_ < .001; Fig 4). These results suggest that the found mechanisms in the present study also exist in an unrelated dataset with highly variable sentence structure.

## Discussion

By focusing on stimulus-specific timescales and measuring comprehension on individual trials, we show that distinct neural and linguistic timescales relate to speech encoding along the auditory–motor pathway. Our findings provide specific functional roles of speech tracking at two timescales within the delta band, one relevant in motor areas—accompanied by modulations in delta–beta coupled oscillations—and one relevant in temporal areas. Previous studies typically show that speech tracking peaks in early auditory areas, independent of the analysed frequency band [2,22,30]. Although a topographical distinction between hierarchical time-scales, as shown in the present data, has been hinted at before [1], this has not been straightforwardly demonstrated.

### The motor system predicts phrasal timing using beta oscillations

The motor system plays a causal role in speech perception [43,44,45]. Previous studies have attributed functions for simulating speech production [46,47] or sensorimotor speech processing [48] to the motor system. Furthermore, the PM cortex and the motor system generally have been associated with generating temporal predictions [49,50,51,52,53] and the processing of rhythms and beats [37,38,39]. In the present study, we increase the knowledge about its role for natural speech processing by uncovering two specific neural mechanisms. The first mechanism is a perceptually relevant speech tracking specifically at the phrasal timescale, peaking in the left PM cortex. Notably, the timing of phrasal elements in the used stimulus corpus was relatively predictable because all sentences followed the same structure. The phrasal structure was also defined by prominent pauses between phrasal elements (evident in the clear peak in the frequency spectrum, S3A Fig). On the other hand, words (and therefore syllables and phonemes) were not semantically—or temporally—predictable due to the recombination of words across sentences. The motor system likely exploited the temporally predictive phrasal information for parsing and segmenting the sentences, thus facilitating comprehension by providing a temporal prediction of when the relevant target word was likely to occur. Our results therefore suggest that perceptually relevant speech entrainment emerges not only at the time-scale of the directly task-relevant feature (here words) but also at those time-scales that can be exploited to better detect or encode this feature. We confirmed this motor mechanism in a second dataset, which featured a less stereotyped phrasal structure. Yet it is possible that this mechanism is not specific to the phrasal structure per se. Instead, it could be that the motor system would exploit any temporal regularities [38], regardless of their linguistic or metalinguistic relevance. Future research is required to directly compare acoustic and linguistic regularities and their relevance for speech tracking.

It has been suggested that delta entrainment to speech in the left hemisphere reflects a motor-driven top-down modulation [42,54]. These top-down modulations have been associated with beta oscillations [50,55,56,57], which are prevalent in the motor system [58]. Beta power in the motor system has also been related to speech comprehension [57]. The finding that the temporal prediction of tone sequences is mediated by prestimulus delta–beta coupled oscillations further supports this hypothesis ([36], see also [40,41]). Here, we show—to our knowledge, for the first time—that such a cross-frequency mechanism also operates during the encoding and perception of continuous speech. This coupling is (i) specific to phrasal delta phase (0.6–1.3 Hz) and beta power (13–30 Hz) and (ii) only perceptually relevant in left motor areas. Furthermore, in an additional dataset, we show that this phrasal delta–beta coupling is also present during the processing of a natural, spontaneous narration. Based on the above-mentioned findings [36,40,41], we speculate that this cross-frequency motor coupling reflects top-down temporal prediction, which is relevant both for the perception of simple sounds [36] and speech.

### Word segmentation in the temporal cortex

Speech tracking at the word scale was perceptually relevant across the entire midtemporal gyrus, peaking in MTG and including superior and inferior temporal gyrus as well as inferior supramarginal gyrus. Previous MEG studies that have localised tracking processes typically show that this peaks in early auditory areas independent of the frequency band (for example, when contrasted with the null hypothesis of no speech encoding) [2,22,30]. Only by using a direct comprehension measure can we show that perceptually relevant word segmentation peaks in the left MTG. The MTG is associated with lexical semantic processes [59,60] and is one endpoint of the ventral auditory pathway, mapping sound to meaning [61]. It is plausible that stronger speech tracking, and therefore better word-scale segmentation in these regions, is directly linked to comprehension performance. The result that the effect at the word scale extends dorsally to supramarginal gyrus seems to contradict models of a ventral focus of word comprehension. However, it is consistent with the notion of a dorsal lexicon, thought to store articulatorily organised word form representations [62].

### Specificity of linguistic timescales

An analysis of 2 Hz–wide generic bands showed that (i) the activity in the motor system was not predictive for comprehension in any generic band; (ii) the 1–3 Hz band, which largely overlaps with the word scale, yielded a similar pattern as the word-specific timescale; and (iii) the 2–4 Hz band also overlapped with the effect at the word timescale, albeit only minimally significantly (S2 Fig). These results suggest that perceptually relevant speech tracking in the motor system is specific to the phrasal timescale in the stimulus material. In temporal regions, perceptually relevant tracking was found in the delta band (above 1 Hz and below 4 Hz), independent of the specific boundaries of the used bands (although 0–2 Hz did not yield a significant effect). This suggests that speech tracking in temporal areas emerges at more widespread timescales, perhaps because word length is more variable than phrasal length in the present stimulus material. Analyses of the coefficient of variation (*c*_v_) supported this interpretation: when compared with phrases (*c*_v_ = 0.27), words varied in length almost twice as much (*c*_v_ = 0.48).

We chose to base the timescales on linguistic categories of phrases, words, syllables, and phonemes. This is the most pragmatic approach because the language system ultimately has to parse the speech stream into these segments. However, one could argue that these linguistic categories overlap with other metalinguistic elements that also follow temporal modulations below 4 Hz such as prosodic features [16]. The most relevant prosodic features for speech segmentation are pauses, stress, and intonation [14,63]. The phrases in the stimulus material are defined by pauses, and therefore phrasal timescale and timing of pauses can be considered one and the same. The interaction between linguistic categories and lexical stress is more complex. If we consider every third syllable as stressed [64], one can derive a ‘stress timescale’ of 0.9 to 1.6 Hz, which partly overlaps with the phrasal timescale (0.6–1.3 Hz). The role of stress is manifold in speech (disambiguation of phonemically identical words, highlighting the meaning of words, metrical stress), and we cannot rule out that stressed syllables are reflected in neural activity. However, the segmentation into phrases does not typically have stressed syllables as boundaries because this would often yield nonsense phrases. Therefore, although stress is important and useful in speech comprehension, focusing on the phrasal timescale (as opposed to the ‘stress timescale’) is a direct way to address phrase segmentation. Fluctuations in pitch, or intonation, also occur in the delta band (see S3B Fig for spectral analysis of pitch, or its acoustic correlate the fundamental frequency). Pitch fluctuations can signal phrasal boundaries [65], and an overlap with the phrasal timescale is therefore not surprising. Because the auditory system is able to track pitch fluctuations [9] and fundamental frequency and intensity are related, we cannot completely disentangle pitch tracking from envelope tracking. But language comprehension requires the grouping of words into phrases [15], and focusing the analysis on the phrasal timescale is the most direct way of analysing phrasal processing. Future research needs to address the question of how much phrasal segmentation relies on the acoustic envelope, pitch fluctuations, or both. Taken together, linguistic and metalinguistic events in natural speech have a tendency to co-occur [66], and their interaction is complex. However, for natural speech processing, the division into linguistic categories, as done in the present study, seems the most pragmatic and ecologically valid solution to gain specificity about speech comprehension effects.

Finally, in the present study, the average speech rate was approximately 130 words per minute. In two other studies that reported speech rate, it was considerably higher, at approximately 160 words per minute [22] and approximately 210 words per minute [25]. The rate of syllables is typically associated with frequencies between 4 and 8 Hz [3,67]. In the present study, it was 2.8 to 4.8 Hz, and in another study, it was even lower at 2 to 4 Hz [4]. These differences demonstrate that, even in experimental contexts, speech rates can deviate from the assumed standard. Furthermore, a recent study has shown that the auditory system is not limited by traditionally imposed frequency bands [57]. It therefore is highly beneficial to calculate stimulus-specific speech regularities for speech tracking analyses instead of applying generic frequency bands (cf. [68] for visual modality).

### Not all speech tracking processes are perceptually relevant

Our results regarding overall speech tracking (compared with chance) replicate previous reports of widespread speech-to-brain entrainment at multiple timescales [2,22]. However, in our data, only speech tracking in specific bands within the delta frequency range differed between trials with correct and incorrect comprehension and was therefore likely relevant for the perceptual outcome. One interpretation of why only the slow timescales were directly perceptually relevant is that the comprehension task focused on words, thus stressing the word timescale. Furthermore, the stereotyped phrasal structure of the sentence provided a temporal structure on which the emergence of the target word could be expected. Therefore, participants may have relied on the encoding of the phrasal structure to exploit its regularity, thereby stressing the phrasal timescale. However, it has been suggested that speech tracking in the delta and theta bands index different functional roles for speech perception [69], such that theta tracking reflects the analysis of acoustic features and delta tracking reflects linguistic representations. In line with the this is the notion that only speech-to-brain entrainment in the delta band reflects active speech-specific processing, as opposed to a passive, low-level synchronisation to acoustic properties at other timescales [30]. Therefore, these and our findings tentatively support the conclusion that only speech tracking in the delta band might indicate a speech-specific, perceptually relevant process during continuous speech processing.

Recent findings also highlight the distinction between widely distributed versus focal (but perceptually relevant) auditory encoding [19,20] that could contribute to this pattern of results. In these accounts, perceptual choices are determined by the efficient readout of a restricted neural area, whereas widespread neural activity represents collateral processes of sensory processing. Therefore, such distributed processes could also explain the widespread speech tracking at all timescales we found when compared to chance level. In the present data, speech tracking at the syllabic and phonetic scales did not differ between trials with correct and incorrect comprehension. But for the participants to comprehend target words correctly, at least some syllables must have been encoded phonetically. It could be that the use of a noisy background prevented the robust encoding of individual syllables or phonemes, thus reducing the tracking at these timescales or reducing the statistical power in detecting between-trial differences. Furthermore, as mentioned above, the use of a word-related task could have highlighted effects at the word level and obscured effects at faster timescales. Additional work is required to understand whether speech tracking at the syllabic and phonetic timescales is indeed a robust marker of the actual neural encoding of these features or whether only speech tracking at timescales below the syllabic rate directly indexes functionally and perceptually relevant processes. Furthermore, the left-lateralised perceptually relevant speech tracking at slow timescales stands in contrast with bilateral overall speech tracking at these scales (Fig 2). This supports the notion that ‘early’ acoustic processes are bilateral, whereas ‘higher-order’ speech comprehension is left-lateralised [70].

## Materials and methods

### Ethics statement

All participants provided written informed consent prior to testing and received monetary compensation of £10 per h. The experiment was approved by a local ethics committee (College of Science and Engineering, University of Glasgow, application number 300140078) and conducted in compliance with the Declaration of Helsinki.

### Participants and data acquisition

Following previous sample sizes of MEG studies that used MI to study speech tracking [2,22], as well as previous recommendations [71,72,73], 20 healthy, native British participants took part in the study (9 female, age 23.6 ± 5.8 years [mean ± SD], age range: 18 to 39 years). All participants were right-handed [Edinburgh Handedness Inventory; 74], had normal hearing [Quick Hearing Check; 75], and normal or corrected-to-normal vision. Furthermore, participants had no self-reported current or previous neurological or language disorders.

MEG was recorded with a 248-magnetometer, whole-head MEG system (MAGNES 3600 WH, 4-D Neuroimaging, San Diego, CA) at a sampling rate of 1 KHz. Head positions were measured at the beginning and end of each run, using 5 coils placed on the participants’ heads. Coil positions were codigitised with head shape (FASTRAK, Polhemus Inc., Colchester, VT). Participants sat upright and fixated at a fixation point projected centrally on screen with a DLP projector. Sounds were transmitted binaurally through plastic earpieces, and 3.7 m–long plastic tubes connected to a sound pressure transducer. Stimulus presentation was controlled with Psychophysics toolbox [76] for MATLAB (The MathWorks, Inc., Natick, MA).

### Stimuli

The stimulus material consisted of 2 structurally equivalent sets of 90 sentences (180 unique sentences in total) that were spoken by a trained, male, native British actor. The speaker was instructed to speak clearly and naturally. Sentences were constructed to be meaningful but unpredictable. Each sentence consisted of the same basic elements and therefore had the same structure. For example, the sentence ‘Did you notice, on Sunday night, Graham offered ten fantastic books’ consists of a ‘filler’ phrase (‘Did you notice’), a time phrase (‘on Sunday night’), a name, a verb, a numeral, an adjective, and a noun. There were 18 possible names, verbs, numerals, adjectives, and nouns that were each repeated 10 times. Sentence elements were randomly combined within a set of 90 sentences. To measure comprehension, a target word was included that was either the adjective in 1 set of sentences (‘fantastic’ in the above example or ‘beautiful’ in Fig 1) or the number in the other set (for example, ‘twenty-one’). The duration of sentences ranged from 4.2 s to 6.5 s (5.4 ± 0.4 s [mean ± SD]). Sentences were presented at a sampling rate of 22,050 Hz.

During the experiment, speech stimuli were embedded in noise. The noise consisted of ecologically valid environmental sounds (traffic, car horns, people talking), combined into a mixture of 50 different background noises. The individual noise level for each participant was determined with a staircase procedure that was designed to yield a performance of around 70% correct. For the staircase procedure, only the 18 possible target words were used instead of whole sentences. Participants were presented with single target words embedded in noise and subsequently saw 2 alternatives on screen. They indicated by button press which word they had heard. Depending on whether their choice was correct or incorrect, the noise level was increased or decreased (one-up-three-down procedure) until a reliable level was reached. The average signal-to-noise ratio across participants was approximately −6 dB.

### Experimental design

The 180 sentences were presented in 4 blocks with 45 sentences each. In each block, participants either indicated the comprehended adjective or the comprehended number, resulting in 2 ‘adjective blocks’ and 2 ‘number blocks’. The order of sentences and blocks was randomised for each participant. The first trial of each block was a ‘dummy’ trial that was discarded for subsequent analysis; this trial was repeated at the end of the block. After each sentence, participants were prompted with 4 target words (either adjectives or numbers) on the screen. They then had to indicate which one they heard by pressing 1 of 4 buttons on a button box. After 2 s, the next trial started automatically. Each block lasted approximately 10 min, and participants could rest in between blocks. The session, including instructions, questionnaires, preparation, staircase procedure, and 4 blocks, took approximately 3 to 3.5 hours.

### Speech preprocessing

For each sentence, we computed the wideband speech envelope at a sampling rate of 150 Hz following procedures of previous studies [2,6,12,77]. Acoustic waveforms were first filtered into 8 frequency bands (between 100 and 8,000 Hz; third-order Butterworth filter; forward and reverse) that were equidistant on the cochlear frequency map [77]. From these 8 individual bands, the wideband speech envelope was extracted by averaging the magnitude of the Hilbert transformed signals from each band.

To define the timescales on which to probe speech encoding, we evaluated the rates of phrases, words, syllables, and phonemes in the stimulus material. For this, the duration between onsets of linguistic categories (i.e., phrases, words, and phonemes) was calculated. The exact onset timing was extracted from the speech signals using Penn Phonetics Lab Forced Aligner (P2FA; [78]). Phrases were defined as the first 2 clauses in each sentence (for example, ‘I have heard’ and ‘on Tuesday night’). These phrases had distinct pauses (see Fig 1 for an example sentence) that determined the rhythm of the sentence (also visible in the frequency spectrum S3A Fig). The syllable rate is generally difficult to assess [18,79]. Here, we chose to count the actually produced syllables for each sentence. Finally, timescales were converted to frequencies, and the specific frequency bands for each category were then defined as the minimum and maximum frequencies across all 180 sentences. This led to the following bands: 0.6–1.3 Hz (phrases), 1.8–3.0 Hz (words), 2.8–4.8 Hz (syllables), and 8–12.4 Hz (phonemes). Mean and standard deviations for linguistic categories were as follows: 1.0 ± 0.1 Hz for phrases, 2.4 ± 0.3 Hz for words, 3.8 ± 0.4 Hz for syllables, and 10.4 ± 0.8 Hz for phonemes. Furthermore, the fundamental frequency for each sentence was extracted using Praat [80]. This was used to determine the frequency spectrum of the pitch fluctuations (see S3B Fig).

### MEG preprocessing

Preprocessing of MEG data was carried out in MATLAB (The MathWorks, Inc., Natick, MA) using the Fieldtrip toolbox [81]. The 4 experimental blocks were preprocessed separately. Single trials were extracted from continuous data starting 2 s before sound onset and until 10 s after sound onset. MEG data were denoised using the reference signal. Known faulty channels (*N* = 7) were removed before further preprocessing. Trials with SQUID jumps (3.5% of trials) were detected and removed using Fieldtrip procedures with a cutoff *z*-value of 30. Before further artifact rejection, data were filtered between 0.2 and 150 Hz (fourth-order Butterworth filters, forward and reverse) and down-sampled to 300 Hz. Data were visually inspected to find noisy channels (4.37 ± 3.38 on average across blocks and participants) and trials (0.66 ± 1.03 on average across blocks and participants). Finally, heart and eye-movement artifacts were removed by performing an independent component analysis with 30 principal components. Data were further down-sampled to 150 Hz to match the sampling rate of the speech signal.

### Source localisation

Source localisation was performed using Fieldtrip, SPM8, and the Freesurfer toolbox. We acquired T1-weighted structural magnetic resonance images (MRIs) for each participant. These were coregistered to the MEG coordinate system using a semiautomatic procedure [2,6]. MRIs were then segmented and linearly normalised to a template brain (MNI space). A volume conduction model was constructed using a single-shell model [82]. We projected sensor-level waveforms into source space using frequency-specific linear constraint minimum variance (LCMV) beamformers [83] with a regularisation parameter of 7% and optimal dipole orientation (singular value decomposition method). Grid points had a spacing of 6 mm, resulting in 12,337 points covering the whole brain.

### Analysis of speech tracking in brain activity

We quantified the statistical dependency between the speech envelope and the source-localised MEG data using MI [2,6,34,84]. The speech envelopes, as well as MEG data, were filtered in the 4 frequency bands reflecting the rates of each linguistic category using third-order (for delta and theta bands) forward and reverse Butterworth filters. Within these bands, we computed the Hilbert transform and used real and imaginary parts for further analysis. Both parts were normalised separately and combined as a two-dimensional variable for the MI calculation [84]. To take into account the stimulus–brain lag, we computed MI at 5 different lags (from 60 to 140 ms in 20-ms steps) and summed the MI values across lags. This procedure prevents spurious results that can occur when using a single lag. First, we calculated the overall MI for each source grid point. For a robust computation of MI values, we concatenated MEG and speech data from all trials. The resulting MI values were compared with surrogate data to determine their statistical significance. Surrogate data were created by randomly shuffling trials 50 times and averaging surrogate MI values across iterations. This repetition was necessary because all sentences followed the same structure and their envelope was often comparable, especially when filtered at low frequencies. We used a dependent *t* test for statistical comparison for each grid point and corrected for multiple comparisons with cluster-based permutation. Specifically, we used Monte-Carlo randomisation with 1,000 permutations and a critical *t* value of 2.1, which represents the critical value of the Student *t* distribution for 20 participants and a two-tailed probability of *p* = .05. The significance level for accepting clusters was 5%. We report summed *t* values (*T*_sum_) as indicator of effect size.

For the analysis of perceptual relevance, we compared MI between trials in which participants responded correctly and incorrectly. Because the number of trials differed between these samples (on average, approximately 70% correct and 30% incorrect), we performed the calculations based on 80% of the minimally available number of trials. This way, the number of compared correct and incorrect trials was equal. However, because this included only a small part of all available trials, we repeated the analysis 20 times with a random selection of trials to yield representative values. The resulting MI values were averaged. Again, trials were concatenated to yield robust MI values. MI values between correct and incorrect trials were compared using the same method and parameters as for the comparison between overall MI and surrogate MI.

To examine the specificity of the effects, we compared MI between correct and incorrect trials for all peak grid points in both frequency bands (i.e., phrasal and word timescales). Peak grid points were those with the largest *t* values in each cluster and the largest summed *t* values for the overlap of grid points. This led to 12 comparisons (3 peak grid points × 4 frequency bands). MI values were compared using dependent sample *t* tests, corrected for multiple comparisons using the FDR method [85].

### PAC

To examine the hypothesis that beta power is coupled with delta phase in the motor cluster and that this is perceptually relevant, we quantified PAC using the MI between beta power and delta phase. Phase and power were derived from Hilbert-transformed time series and filtered in the phrasal (0.6–1.3 Hz) and beta band (13–30 Hz). Phase was expressed as a unit magnitude complex number. To get an equal number of trials for correct and incorrect trials, we again took 80% of trials of the smaller sample, concatenated trials, and repeated the calculation 50 times. This was done for all grid points within the motor cluster (*N* = 205) and then averaged across grid points and iterations. PAC was compared between correct and incorrect trials across participants using a dependent sample *t* test.

We performed 3 control analyses within the motor cluster to address the frequency specificity of the effect. First, we analysed PAC between phrasal phase (0.6–1.3 Hz) and alpha power (8–12 Hz) as well as theta power (4–8 Hz). Second, we analysed PAC between the word phase (1.8–3 Hz) and beta power. All *p*-values were corrected for multiple comparisons using the FDR method [85].

To address the spatial specificity of the delta–beta PAC, we also performed a whole-brain analysis. Based on the results in the motor cluster, we hypothesised that PAC should be larger in correct than incorrect trials. PAC between phrasal delta phase (0.6–1.3 Hz) and beta power (13–30 Hz) was compared between correct and incorrect trials, again equalling sample sizes by using 80% of the minimally available number of trials and repeating the analysis 20 times. PAC MI was averaged across all iterations and then compared between correct and incorrect trials across participants using a dependent sample *t* test for each grid point. To correct for multiple comparisons, we used the same parameters for cluster correction as in all previous analyses except that the significance level to choose significant clusters was one-sided, due to the clear hypothesis.

### Analysis of a previously published dataset

We analysed an additional and previously published dataset to confirm the present effects in the motor cortex. For this, we used data from 23 participants [2,42] who passively listened to a 7-min continuous natural narration. Preprocessing and analysis were identical to the procedures of the main data. We compared (i) phrasal tracking and (ii) PAC between phrasal phase and beta power with surrogate data in the motor cortex. The phrasal rate of the speech stimulus was 0.5 ± 0.26 Hz (mean ± SD) and ranged between 0.1 Hz and 1.5 Hz. Surrogate data were created by reversing the time series for speech and computing MI between forward brain time series and reversed speech time series. This represents values that would be expected by chance [2]. Values were computed for all grid points in the motor cluster and then spatially averaged. Actual MI values and surrogate data were compared using a dependent *t* test, and *p*-values for both tests were FDR corrected.

Data were deposited in the Dryad repository (https://doi.org/10.5061/dryad.1qq7050) [31].

## Supporting information

S1 Fig Comparison of MI values at peak grid points in PM gyrus, MTG, and HG for syllable and phoneme scales. Boxes denote interquartile range with median line; error bars show minimum and maximum, excluding outliers. None of the comparisons reached significance (all *p*_FDR_ > .56, *p*_uncorrected_ > .20). Data deposited in the Dryad repository: https://doi.org/10.5061/dryad.1qq7050 [31]. FDR, false discovery rate; HG, Heshl gyrus; MI, mutual information; MTG, middle temporal gyrus; PM, premotor.
(TIF)

S2 Fig Analyses in generic 2 Hz–wide bands. Seven overlapping frequency bands were analysed (from 0–8 Hz, in 2 Hz–wide bands, in 1-Hz steps). The first 3 of these bands are displayed here. (A) Perceptually relevant tracking (larger MI for correctly comprehended than incorrectly comprehended trials) was found at the 1–3 Hz scale (*T*_sum_(19) = 1,078.85, *p*_cluster_ = .030) and at the 2–4 Hz scale (*T*_sum_(19) = 751.93, *p*_cluster_ = .046). Effects in all other bands were *p* > .11. (B) Analysis of the peak grid points that showed the strongest effect in stimulus-specific bands. Larger MI for correctly than incorrectly comprehended trials is found in HG in the generic 1–3 Hz band (*t*(19) = 4.54, *p*_FDR_ = .002) and in MTG in the 2–4 Hz band (*t*(19) = 3.38, *p*_FDR_ = .014). All other comparisons are *p* > .08. The peak grid point in the PM cortex does not show a comprehension modulation in any of the generic bands. Data deposited in the Dryad repository: https://doi.org/10.5061/dryad.1qq7050 [31]. FDR, false discovery rate; HG, Heschl gyrus; MTG, middle temporal gyrus; PM, premotor; *T*_sum_, summed *t* values.
(TIF)

S3 Fig Power spectral density estimates of speech envelope and pitch. Welch’s periodograms are shown for speech envelopes (A) and fundamental frequency (F0-contours/pitch) (B) of all 180 stimulus sentences (thin gray lines) and their average (thick black line), for frequencies between 0.1 and 12 Hz (in 0.1-Hz steps). For envelope spectra, visible peaks that correspond to rates used in the analysis are marked with arrows (i.e., for phrases, words, and—less pronounced—syllables). Data deposited in the Dryad repository: https://doi.org/10.5061/dryad.1qq7050 [31].
(TIF)

## Abbreviations

BA: Brodmann area
*c*_v_: coefficient of variation
FDR: false discovery rate
HG: Heschl gyrus
LCMV: linear constraint minimum variance
MEG: magnetoencephalography
MI: mutual information
MRI: magnetic resonance image
MTG: middle temporal gyrus
n.s.: not significant
PAC: phase-amplitude coupling
PM: premotor
*T*_sum_: summed *t* values
