# Supplementary figures and images for: Perceptually relevant speech tracking in auditory and motor cortex reflects distinct linguistic features
Source: PLoS Biol. 2018 Mar 12;16(3):e2004473. doi: 10.1371/journal.pbio.2004473 (PMC5864086; doi:10.1371/journal.pbio.2004473)

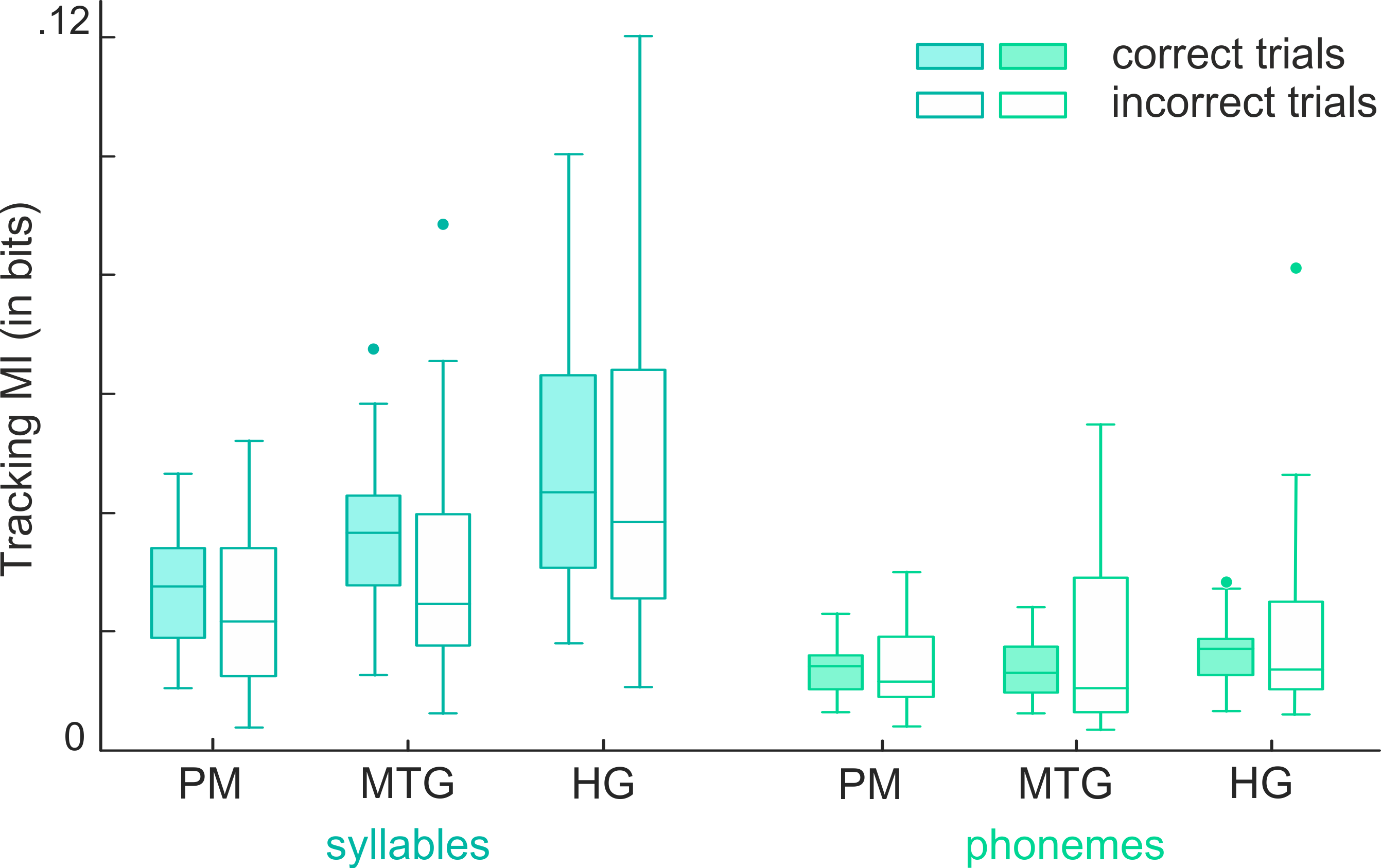

Supplement: S1 Fig — Boxes denote interquartile range with median line; error bars show minimum and maximum, excluding outliers. None of the comparisons reached significance (all pFDR > .56, puncorrected > .20). Data deposited in the Dryad repository: https://doi.org/10.5061/dryad.1qq7050 [31]. FDR, false discovery rate; HG, Heshl gyrus; MI, mutual information; MTG, middle temporal gyrus; PM, premotor. (TIF) [file pbio.2004473.s001.tif]

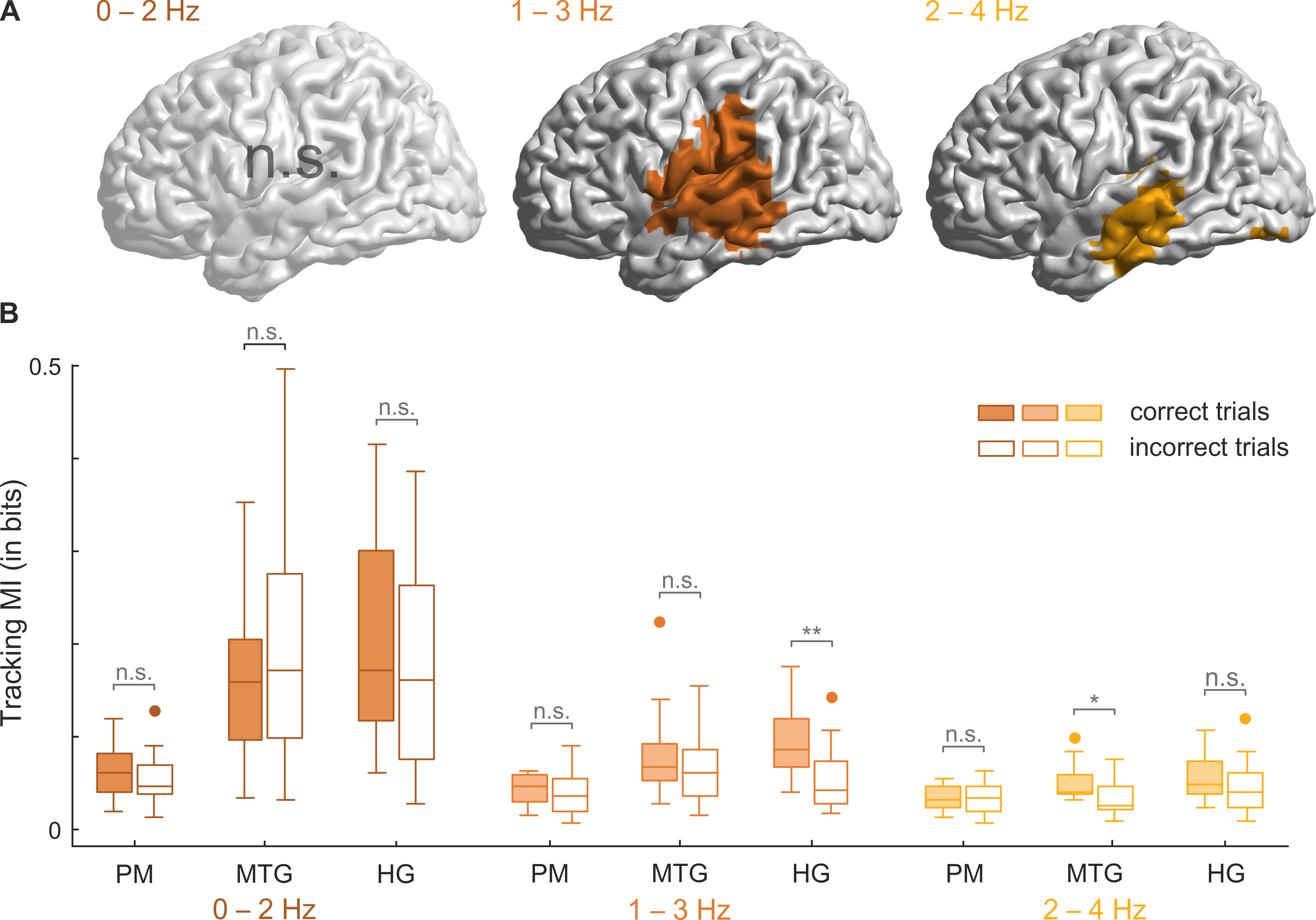

Supplement: S2 Fig — Seven overlapping frequency bands were analysed (from 0–8 Hz, in 2 Hz–wide bands, in 1-Hz steps). The first 3 of these bands are displayed here. (A) Perceptually relevant tracking (larger MI for correctly comprehended than incorrectly comprehended trials) was found at the 1–3 Hz scale (Tsum(19) = 1,078.85, pcluster = .030) and at the 2–4 Hz scale (Tsum(19) = 751.93, pcluster = .046). Effects in all other bands were p > .11. (B) Analysis of the peak grid points that showed the strongest effect in stimulus-specific bands. Larger MI for correctly than incorrectly comprehended trials is found in HG in the generic 1–3 Hz band (t(19) = 4.54, pFDR = .002) and in MTG in the 2–4 Hz band (t(19) = 3.38, pFDR = .014). All other comparisons are p > .08. The peak grid point in the PM cortex does not show a comprehension modulation in any of the generic bands. Data deposited in the Dryad repository: https://doi.org/10.5061/dryad.1qq7050 [31]. FDR, false discovery rate; HG, Heschl gyrus; MTG, middle temporal gyrus; PM, premotor; Tsum, summed t values. (TIF) [file pbio.2004473.s002.tif]

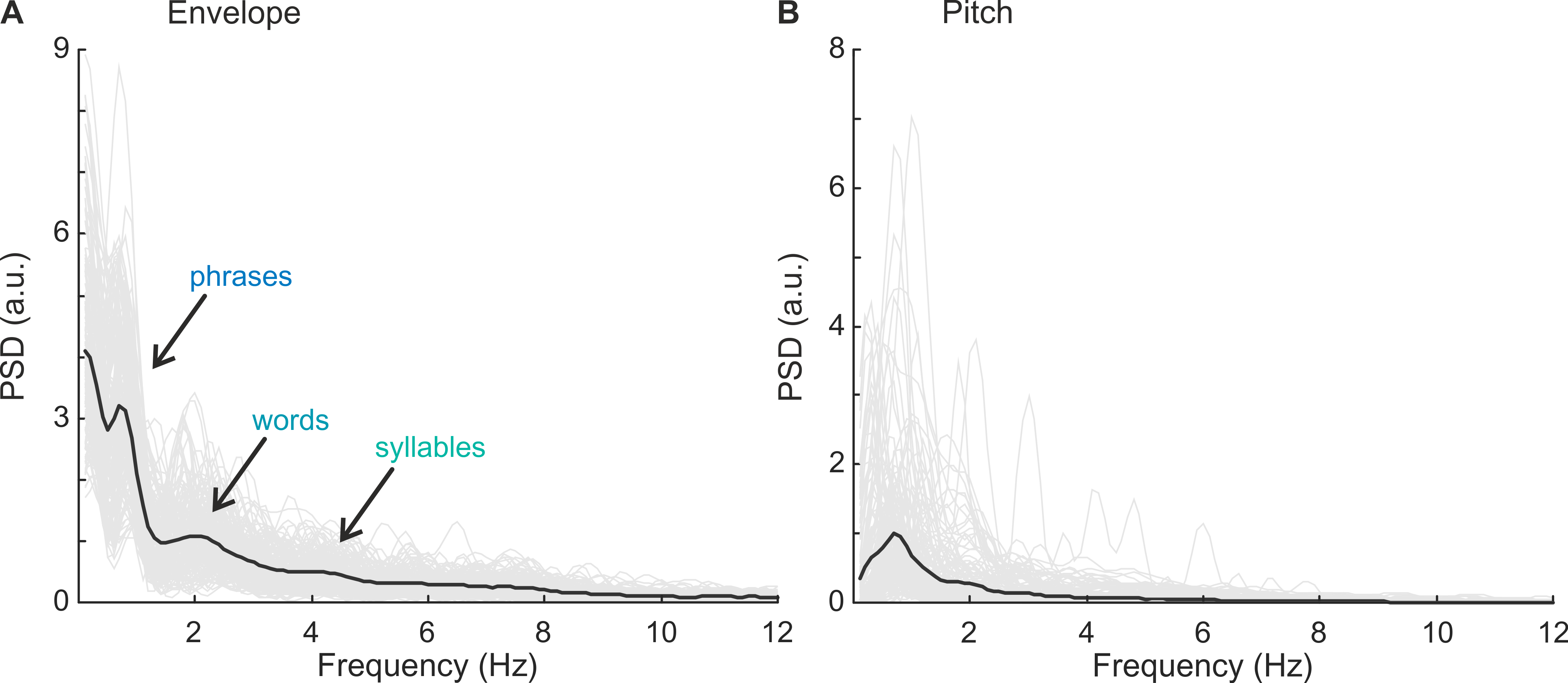

Supplement: S3 Fig — Welch’s periodograms are shown for speech envelopes (A) and fundamental frequency (F0-contours/pitch) (B) of all 180 stimulus sentences (thin gray lines) and their average (thick black line), for frequencies between 0.1 and 12 Hz (in 0.1-Hz steps). For envelope spectra, visible peaks that correspond to rates used in the analysis are marked with arrows (i.e., for phrases, words, and—less pronounced—syllables). Data deposited in the Dryad repository: https://doi.org/10.5061/dryad.1qq7050 [31]. (TIF) [file pbio.2004473.s003.tif]
